# One-pot strategy to simultaneously prepare dyed, flame retardant and UV-resistant silk fabric based on a safflower yellow derivative

**DOI:** 10.1039/d2ra04873f

**Published:** 2022-09-15

**Authors:** Dan Yu, Yansong Liu, Yuanlin Ren, Xiaohui Liu, Hongqiang Qu

**Affiliations:** School of Textile Science and Engineering, Tiangong University Tianjin 300387 China yuanlinr@163.com +86-22-8395-8287 +86-22-8395-5353; School of Materials Science and Engineering, Tiangong University Tianjin 300387 China xiaohuilau@163.com; College of Chemistry and Environmental Science, Hebei University Baoding 071002 China hqqu@163.com

## Abstract

To give silk fabric multiple functions and improve its application range, a facile and eco-friendly one-pot technique was used to endow silk fabric with dyeing, flame retardant and ultraviolet (UV)-resistant performance based on a safflower yellow derivative. The structure, surface morphology, elemental composition, thermal properties and combustion behavior of the silk samples before and after modification were all characterized. In addition, the color depth and color fastness of the dyed silk (D-silk) and flame retardant silk (FR-silk) were evaluated and measured. The positive b* value in the CIELAB color space defined the yellow color of the dyed silk fabric. The char residue increased from 2.9 wt% of the original sample to 17.4 wt% of the FR-silk at 800 °C in air atmosphere. The limiting oxygen index (LOI) of FR-silk increased from 23.3% of the control sample to 32.8% with an increase of 40.7%. The peak of heat release rate (pHRR) and total heat release (THR) of FR-silk were much lower than that of the original sample. Furthermore, the UV protection factor (UPF) of FR-silk was 73.79, indicating excellent UV-resistance properties. The preparation process was green and environment-friendly, and the FR-silk had a good application prospect.

## Abstract 2

A facile and eco-friendly one-pot technique was used to endow silk fabric with dyeing, flame retardant and ultraviolet (UV)-resistant performance based on a safflower yellow derivative.

## Graphical abstract

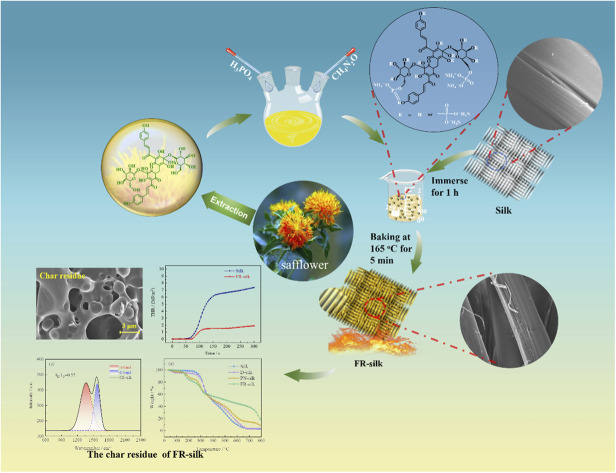

## 1. Introduction

Silk enjoys a reputation of the second skin of human beings due to its good hygroscopicity, anti-ultraviolet and biocompatibility with human body.^1,2^ As a natural material, silk is widely used in bedding, decorative items and clothing. However, silk fabric catches fire easily due to its low limiting oxygen index (LOI) value, which limits its wider application. Thus, it is necessary to improve its flame retardant properties.^3^ For commercial flame retardants, halogen-containing flame retardants are the most used flame retardants because of their excellent flame retardancy. However, some halogen-based flame retardants are considered carcinogenic, and may cause environmental pollution and endanger human health. Hence, they have been gradually restricted or even forbidden in recent years.^4^ Therefore, researchers are gradually focusing their attention on natural compounds or other non-toxic flame retardants.^5^

To date, various available and sustainable methods have been developed to enhance the flame resistance of silk fabric. Sol–gel, UV grafting and finishing process are often used to endow silk with flame retardancy.^6–8^ For example, a new ternary flame retardant sol system based on tetraethyl orthosilicate as a precursor and boric acid and urea as flame retardant additives was developed and applied to silk fabric. The results showed that the sol was successfully coated on the silk surface, and the thermal stability and smoke suppression performance of the treated silk fabric were better than that of the control sample.^9^ Guan *et al.* treated silk with dimethyl-2-(methacryloyloxyethyl) phosphate to prepare flame retardant silk. Compared to the original sample, the decomposition temperature of the modified silk decreased by 41 °C.^10^ A boron-containing flame retardant was applied to silk fabric to improve its flame retardant performance. The LOI of the treated silk fabric was greater than 28% and had self-extinguishing even after five laundering cycles (LCs).^11^ Nowadays, the development of silk fabric with multifunctionality, such as flame retardant and antibacterial, flame retardant and UV resistance, has become a research hotspot. For example, Zhou *et al.* used laccase and natural polyphenols to treat silk fabric followed by dopamine iron modification. The resulted fabric not only had excellent hydrophobic function, but its UV protection factor (UPF) value could reach 72, exhibiting excellent UV shielding property. In addition, the vertical burning test showed that the damaged length of the burnt fabric was only 10.5 cm, while the LOI value could reach 28%.^12^ The development of potential flame retardant components extracted from plants and their application in silk fabrics has attracted great attention of researchers. Zhang *et al.* used tannic acid and ferrous ion complex to modify silk. The LOI of the modified silk fabric got 27.5%, and the antibacterial efficiency remained 90% after 20 LCs.^13^ Cheng *et al.* extracted active ingredients from tea straw waste under alkaline conditions and applied them to silk fabric to impart silk fabric excellent flame retardant and dyeing properties. However, the tensile strength of the modified silk fabric decreased slightly.^14^ Guo *et al.* used proanthocyanidins extracted from grape seed to endow silk fabrics with flame retardancy, dyeing and antibacterial functions. The flame retardant and antibacterial properties were durable, but the light resistance was relatively worse.^15^ Safflower yellow (SY) has many advantages, such as antioxidant, non-toxic, and can be used for the therapy of heart disease.^16,17^ SY, as a sustainable resource, can be used as natural dye and reduce the use of chemically synthesized dyes to a certain extent and meet the requirement of environmental protection. In addition, SY has excellent compatibility with silk fabric, which is conducive to dyeing. In this work, a dyed, flame retardant and UV-resistant silk fabric was fabricated based on safflower yellow derivative through a facile one-pot strategy. The surface morphology, structure, thermal behavior, combustion, color fastness and UV-resistant properties of the silk fabric before and after modification were all investigated. The modified silk fabric had an excellent flame retardancy and good color fastness. In addition, the treated silk fabric had satisfied UV protection. Meanwhile, this fabrication process was not only convenient and practical but also economically and environmentally friendly.

## 2. Experimental section

### 2.1. Materials

Pure silk fabrics (81 g m^−2^) were purchased from Alibaba (Tianjin, China). The silk fabrics were washed with anhydrous ethanol at room temperature for 10 min and ultrasonically cleaned with distilled water before modification. Safflower yellow (SY) was supplied by Nantong Tianxiang Biological Engineering Co. Ltd (Nantong, China). Phosphorus acid (85%), anhydrous ethanol and urea were obtained from Tianjin Guangfu Fine Chemical Research Institute (Tianjin, China). Dicyandiamide was provided by Tianjin Kermel Chemical Reagent Co. Ltd (Tianjin, China). Distilled water was provided by the Textile Laboratory of Tiangong University (Tianjin, China). All reagents were used as received.

### 2.2. Preparation of ammonium salt of SY (ASY)

**Fig. 1:**
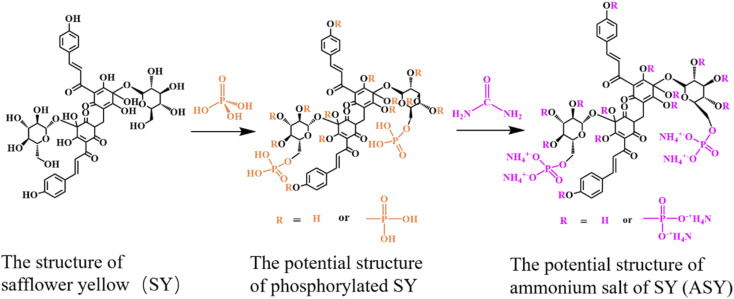
ossible synthesis route of ASY.

SY (3.0 g) and 100 mL of distilled water were mixed in a 250 mL three-necked flask equipped with a reflux condenser and a magnetic stirrer. Afterwards, phosphoric acid (85%, 5.8 g) mixed with 25 mL of distilled water was poured into the flask. After 1.5 h, the rection was heated to 120 °C in an oil bath, and urea (3.0 g) dissolved in 25 mL of distilled water was slowly added to the solution. The reaction was kept at 120 °C for 0.5 h. Next, the reaction mixture was cooled to ambient temperature, and poured into a beaker containing 250 mL of anhydrous ethanol. The mixture was refrigerated at 0 °C for 12 h and filtered under reduced pressure to obtain a yellow solid, *i.e.* ammonium salt of SY (ASY). Fig. 1 illustrated the possible synthesis route of ASY.

### 2.3. Fabrication of flame retardant silk (FR-silk)

ASY (3.0 g), dicyandiamide (10.5 g) and distilled water (100 mL) were mixed in a 250 mL flask equipped with a reflux condenser and a magnetic stirrer. The silk fabrics were soaked into the ASY solution with a bath ratio of 1 : 50 at 90 °C for 1 h. Then, the samples were squeezed to ensure the liquid retention of 150%. Next, the silk fabrics were cured at 165 °C for 4 min. Finally, the samples were washed with distilled water and dried at 60 °C in an oven to a constant weight, *i.e.*, the flame retardant silk (FR-silk) was prepared.

### 2.4. Fabrication of dyed silk fabric (D-silk)

SY (3.0 g) and distilled water (100 mL) were mixed in a 250 mL flask equipped with a magnetic stirrer. The silk fabric was soaked into the solution with a bath ratio of 1 : 50 at 90 °C for 1 h. Then the silk fabric was taken out and squeezed to make the liquid retention of 150%. After that, the treated sample was washed with distilled water and dried at 60 °C in an oven to a constant weight. Finally, the dyed silk fabric (D-silk) was obtained.

### 2.5. Fabrication of phosphorylated silk fabric (PN-silk)

Phosphoric acid (85%, 5.8 g) and urea (3.0 g) dissolved in 150 mL distilled water were added to a 250 mL three-necked flask equipped with a reflux condenser and a magnetic stirrer. The rection was heated to 120 °C for 2 h. Then, dicyandiamide (10.5 g) was dissolved in the reaction solution in a 250 mL flask equipped with a magnetic stirrer and added. The silk fabric was immersed in the aforementioned solution with a bath ratio of 1 : 50 at 90 °C for 1 h. Next, the silk fabric was removed and gently squeezed to maintain 150% liquid retention. Afterwards, it was baked in an oven at 165 °C for 4 min. The treated samples were washed with distilled and dried at 60 °C in an oven to a constant weight, and the phosphorylated silk fabric (PN-silk) was obtained.

The weight gains (WG) of the treated silk fabrics were calculated using the following formula:WG (wt%) = [(*m*_2_ − *m*_1_)/*m*_1_] × 100%where, *m*_1_ and *m*_2_ represent the weight of the original and treated sample, respectively. In this work, PN-silk with a WG of 5.8 wt% and FR-silk with a WG of 8.2 wt% were used for the following experiments.

### 2.6. Characterizations

X-ray photoelectron spectroscopy (XPS) was carried out on a PH15300 spectrometer to characterize the elemental composition of all the silk fabrics.

Thermogravimetric analysis (TGA) was recorded on a STA449F3 thermogravimetric instrument (Netzsch, Germany). The testing samples were heated from ambient temperature to 800 °C with a heating rate of 10 °C min^−1^ in air or nitrogen atmosphere, respectively.

The structure of all the samples was measured by Fourier transform infrared spectroscopy (FTIR) in the range of 500 and 4000 cm^−1^ with 2 cm^−1^ resolution.

Cone calorimetry tests of pure and flame retardant silk samples were performed on an FTT0006 cone calorimeter (FTT, UK) to characterize the combustion properties according to ISO 5660-1 with an irradiation heat flow of 35 kW m^−2^.

Raman spectra of the residue char of samples were recorded on a laser Raman instrument (XploRA PLUS, Japan) in the range of 500–2500 cm^−1^.

The LOI values of samples were determined by a M606B digital oxygen index apparatus (Qingdao Shanfang Instrument Co., Ltd, Shandong, China) according to ASTM D2863-2000 standard.

Burning tests were carried out according to GB/T 5455-1997 standard to compare the burning properties of pure and treated samples with a size of 2 cm × 8 cm.

The color characteristics (L*, a*, b* and *K*/*S*) of FR-silk were obtained at a 10° field of view using a Datacolor-800 (DataColor Technology Co., USA) D65 light source. The GB/T 3921-2008 standard was used for detecting the color fastness and flame retardancy to washing, with 45 min being a laundering cycle and the temperature was set at 50 °C.

The ultraviolet (UV) resistance performance of silk fabrics before and after modification were tested by YG909-III Fabric UV tester (Fangyuan Instrument Co., Ltd, Zhejiang, China) based on GB/T 18830-2002.

The tensile strength and breaking elongation of different silk fabrics were tested on YG065H250/pc electronic fabric strength machine according to GB/T3923.1-1997 standard.

## 3. Results and discussion

### 3.1. Structural characterization of ASY

**Fig. 2:**
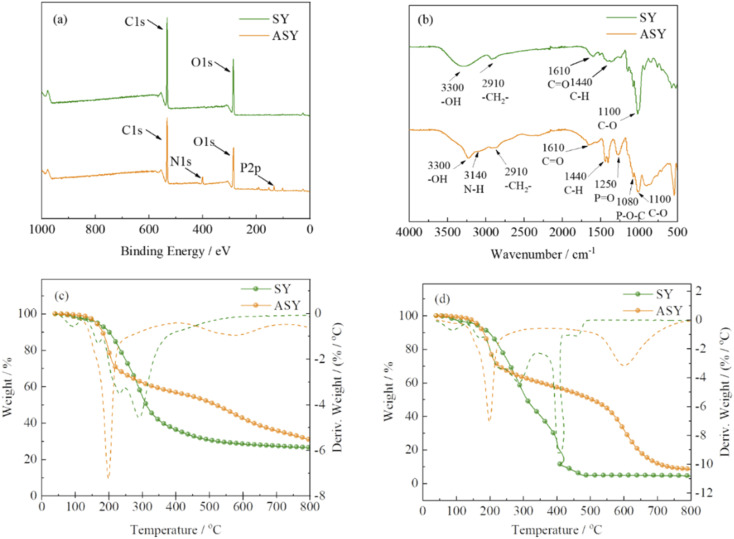
The XPS spectra (a) of SY and ASY; FT-IR spectra (b) of SY and ASY; TG and DTG curves of SY and ASY in nitrogen (c) and air atmosphere (d).

The structure of ASY was measured by XPS and FT-IR. The chemical composition of ASY was proved by XPS and its corresponding spectra was displayed in Fig. 2a. For SY, the peaks at 285, 531 eV indicated the presence of C1s and O1s, respectively. Compared to SY, two new peaks at 134 and 400 eV were assigned to P2p and N1s for ASY. This revealed the successful introduction of P and N elements in ASY. As shown in Fig. 2b, the obvious absorption peaks at 1110, 1440 and 2910 cm^−1^ were assigned to the C–O, C–H and –CH_2_ of SY, respectively.^18,19^ The absorption peak at 1610 cm^−1^ was ascribed to the stretching vibrations of C=O group. The peak at 3300 cm^−1^ was attributed to the stretching vibration of –OH both in SY and ASY. Compared with SY, the peak pattern of ASY changed from broad and smooth to narrow and sharp, which demonstrated that the highly reactive –OH of SY was phosphorylated. The spectra of ASY exhibited new peaks at 1250 cm^−1^ and 3140 cm^−1^ corresponding to P=O and N–H,^20^ which were derived from the ammonium phosphate groups in ASY. In addition, the new characteristic absorption peak of ASY at 1080 cm^−1^ was due to P–O–C, which was the evidence for the successful synthesis of ASY. In short, ASY was successfully synthesized as expected.

The thermal properties of SY and ASY were evaluated by thermogravimetric (TG) analysis. Fig. 2c and d exhibited the TG and DTG curves of SY and ASY in nitrogen and air condition, respectively. In nitrogen atmosphere (Fig. 2c), SY was divided into two main thermal degradation stages. The first weight loss stage occurred at around 100 °C, which was mainly due to the evaporation of the absorbed water. The second one began from 120 to 410 °C, which was attributed to the cyclization, crosslinking and carbonization decomposition between the benzene ring and its substituents. As a result, the char residue of SY was only 26.5%. For ASY, the main weight loss stage occurred between 198 and 334 °C because of the release of small molecular phosphorus containing acids. The char residue of ASY at 800 °C was up to 31.1 wt%, showing excellent thermal stability. In air condition, for SY, due to further oxidation effect at high temperature, only 4.5 wt% char residue was left. As far as ASY was concerned, the first thermal weight loss was similar to that in nitrogen, but the second thermal weight loss in air is greater than that in nitrogen, which is due to the further oxidative decomposition of the generated char. The char residue of ASY at 800 °C was lower than that in nitrogen, but higher than SY. It was concluded that the high temperature thermal oxidation stability of ASY provided a prerequisite for its application as a flame retardant modifier for silk.

### 3.2. Elemental analysis of the fabric

**Fig. 3:**
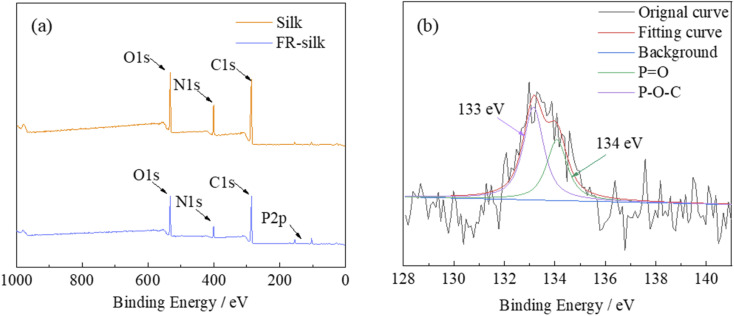
XPS spectra of the control and FR-silk fabric.

The elemental compositions of pure and FR-silk fabrics were studied by XPS, as shown in Fig. 3a. The relevant data were summarized in Table 1. For the control and FR-silk samples, the peaks corresponding to C1s, O1s and N1s appeared at 285, 531 and 400 eV.^21^ As shown in Fig. 3a, FR-silk showed a absorption peak at 134 eV ascribing to P element, which suggested that ASY was successfully introduced into the silk matrix.^22^Fig. 3b presented the P2p spectra of FR-silk. The peak at 133 eV corresponded to P–O–C, which was derived from the binding bond between ASY and silk fabric. In addition, the peak located at 134 eV was assigned to the unreacted –P=O(O–NH_4_^+^)_2_ groups.^23,24^ Compared with the original fabric, the C, O and N contents of FR-silk changed little. However, the P content of FR-silk fabric was nearly three times of the control sample, suggesting that P and N elements were successfully incorporated into silk fabric, which was conducive to the improvement of flame retardancy of FR-silk.

**Table 1:** Element contents of the control and FR-silk

Sample	Element contents (at%)			
	C	O	N	P
Silk	47.83	30.80	20.88	0.49
FR-silk	48.15	30.21	20.18	1.45

### 3.3. FT-IR analysis

**Fig. 4:**
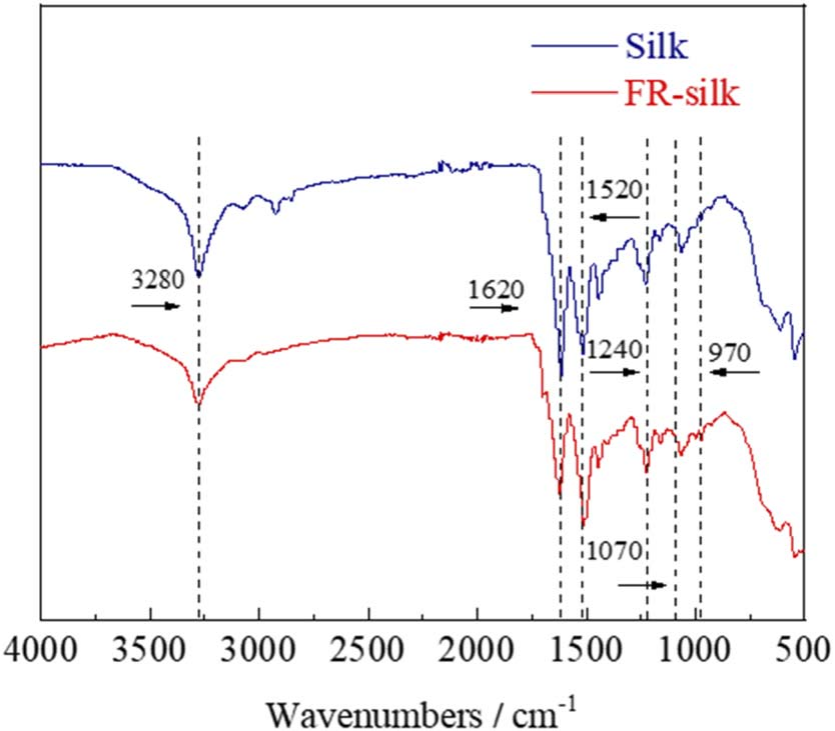
FTIR spectra of pure silk and FR-silk.

The chemical structures of the control and FR-silk were characterized by FTIR, as shown in Fig. 4. The peaks at 1620, 1520 and 1240 cm^−1^ were assigned to C=O of amide I, N–H of amide II and C–H of amide III for the control and FR-silk samples, respectively.^25^ In addition, the characteristic peaks of P=O and P–O–C appeared at 1070 and 970 cm^−1^ in FR-silk spectrum, indicating the successful introduction of phosphorus contents. The intensity of –OH absorption peak at 3280 cm^−1^ of FR-silk was slightly weaker than that of blank sample, which was attributed to the effective reaction between ASY and –OH in silk molecule.^26,27^ The FTIR results further demonstrated that ASY was successfully bound to silk fabric, which was consistent with the XPS results. That was to say that ASY did react with the silk fabric, ensuring silk fabric good flame retardancy.

### 3.4. Morphological analysis

**Fig. 5:**
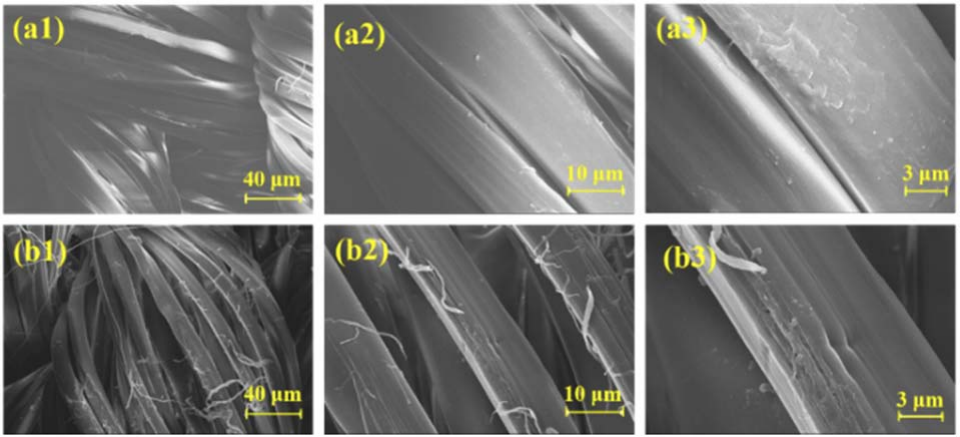
SEM images of the control silk (a1, a2, a3) and FR-silk (b1, b2, b3).

The surface morphology of silk fabric before and after modification was observed by SEM. As shown in Fig. 5a1–a3, the fiber surface of the blank sample was smooth and textured and the fibers were closely wrapped each other. For FR-silk, as indicated in Fig. 5b1–b3, there were obvious grooves and some fine particles on the fiber surface. The weak acidity of ASY aqueous solution led to the partial hydrolysis of amide bonds in silk molecular, forming grooves with different depths on the fiber surface.^28^ The grooves increased the specific surface area of the fiber, so that ASY molecules could easily penetrate into the fabric and bond with the fiber surface, so as to significantly improve the color fastness and flame retardant durability of FR-silk.

### 3.5. Thermal stability analysis

**Fig. 6:**
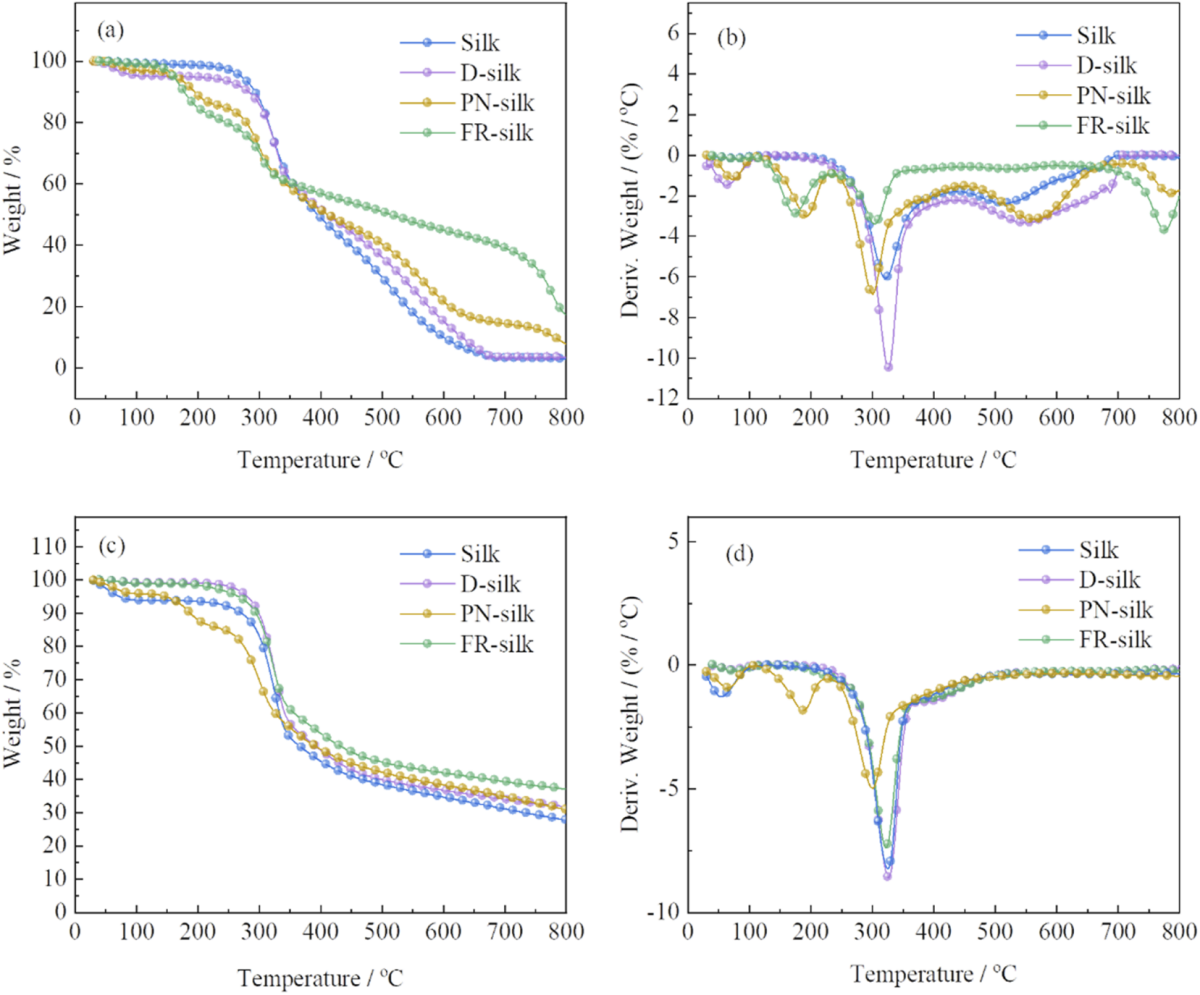
TG and DTG curves of silk, D-silk, PN-silk and FR-silk in air (a and b) and nitrogen atmosphere (c and d).

In order to evaluate the thermal stability of the silk before and after modification, the thermogravimetric analysis (TGA) and derivative thermogravimetric (DTG) were performed in nitrogen and air atmosphere, as shown in Fig. 6 and Table 2. In air atmosphere, the control sample exhibited three weight loss stages. The first one was between 40 and 100 °C with a weight loss of about 3 wt%, which was due to the vaporization of the water attached to silk fabric.^29^ The second one ranged from 200 to 400 °C with 49.81 wt% weight loss, owing to the further pyrolysis of the degraded products and destruction of the internal structure of silk.^30^ During the third stage, with the temperature increasing, the char residue continued to thermal oxidative decompose and produce more CO_2_. Thus, the char residue of silk at 800 °C was only 2.9 wt%. For D-silk, the weight loss before 150 °C was attributed to the evaporation of the bounded water. However, the main thermal degradation took place between 260 and 352 °C, which was delayed compared to the silk sample. This was mainly due to the fact that after the silk fabric was dyed with saffron yellow, the polyhydroxyl structure conferred the potential thermal stabilization of silk fabric.

**Table 2:** TG and DTG data of the control and modified silk fabrics in nitrogen and air atmosphere

Atmosphere	Samples	*T*_5%_ (°C)	*T*_50%_ (°C)	*T*_max%_ (°C)	Residue at 800 °C (wt%)
Air	Silk	269	393	321	2.9
	D-silk	195	411	326	3.7
	PN-silk	164	410	298	8.1
	FR-silk	160	515	775	17.4
N_2_	Silk	281	392	326	27.8
	D-silk	201	266	323	31.1
	PN-silk	149	393	299	30.9
	FR-silk	266	432	321	37.1

In order to assess the effect of phosphorylation on the improvement of the thermal stability of silk fabric in this work, the thermal property of silk fabric treated only with phosphoric acid and urea (PN-silk) was studied. The thermal decomposition curve of PN-silk was similar to that of FR-silk before 350 °C. The thermal weight loss of the two fabrics in this stage was attributed to the removal of the bounded water and the early decomposition of phosphorus containing groups. However, when the temperature was higher than 350 °C, the thermal stability of FR-silk was significantly higher than that of PN-silk, and the thermal weight loss was slower than that of PN-silk. This was closely related to the chemical structure of ASY and the robust chemical bonds between ASY and the fabric. At the same time, compared with PN-silk, the excellent thermal stable benzene ring in ASY was helpful to improve the thermal stability of silk. In a word, although PN-silk showed an improvement in thermal stability, the improvement was far lower than that of FR-silk.

Compared to the original silk, FR-silk showed four weight loss stages due to the effect of flame retardant. The first one was similar to that of silk sample, ascribing to the loss of free water attached to FR-silk matrix. The second one was between 100 and 250 °C with a weight loss of 19.4 wt%, which was much earlier than that of the second thermal decomposition stage of pure sample, corresponding to the advanced decomposition (176 °C) of the ASY introduced into the silk matrix. At this stage, ASY was decomposed to generate phosphorus-containing components such as phosphoric acid, pyrophosphoric acid, and polyphosphoric acid which catalyzed the dehydration of silk into char. The next stage ranged from 250 to 400 °C with about 19.87 wt% weight loss, which was much lower than that of silk sample. The acidic groups generated by the second stage promoted the char formation of fiber matrix and increased the thermal stability of fiber. Due to the high thermal stability of the char layer formed by phosphorous-containing acid catalyzation, the weight loss in the fourth stage occurred at 700–800 °C, at the same time, the char residue was further oxidized to generate CO_2_, leaving a char residue of 17.8 wt%. Furthermore, char the residue of FR-silk was approximately twice as high as that of PN-silk at 800 °C. The thermal degradation of FR-silk was remarkably reduced after 350 °C compared to PN-silk.

In nitrogen atmosphere, the thermal decomposition behavior of the control sample was similar to that of PN-silk below 150 °C, which was due to the evaporation of water from the silk fabric.^31^ However, the thermal decomposition behavior of D-silk was similar to that of FR-silk before 230 °C. PN-silk started to decompose at around 160 °C, while FR-silk showed better thermal stability at this stage. The main weight loss of the four samples was between 250 and 386 °C, which was due to the breakdown of peptide bonds, destruction of side chain groups of amino acid in silk fabric and the decomposition of phosphorus-containing groups. At this stage, the weight loss of PN-silk was lower than that of the other three samples, demonstrating that phosphorus-containing groups in silk greatly inhibited the thermal decomposition of silk and catalyzed the char formation of silk. Compared to PN-silk, the weight loss of FR-silk was greater at this stage, however, the char residue of FR-silk was significantly up to 37.1 wt% at 800 °C, which was higher than the other three samples. This suggested that ASY effectively promoted the char-forming ability of silk.^32^ The results revealed that the thermal stability of D-silk was significantly higher than that of pure silk, which proved that SY had positive influence on improving the thermal stability of silk. It was worth noting that although the thermal stability of D-silk was improved, the flame retardant performance was not improved. Therefore, dyeing silk with ASY was highly feasible to improve the thermal stability and flame retardancy of silk.

### 3.6. Combustion properties

**Fig. 7:**
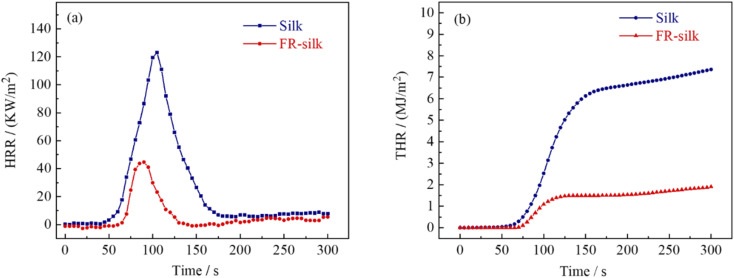
HRR (a) and THR (b) curves of the control and FR-silk.

The peak of heat release rate (pHRR) was considered as one of the most important parameters to assess the combustion performance of materials. As shown in Fig. 7a and Table 3, the pHRR of silk fabric was at 105 s with a value of 123.2 kW m^−2^. However, the pHRR of FR-silk was at 90 s with a value of 44.7 kW m^−2^ and 63.71% reduction. The total heat release (THR) of FR-silk fabric decreased from 7.3 MJ m^−2^ of the control sample to 1.9 MJ m^−2^ with about 74% reduction (Fig. 7b). Fire growth rate index (FIGRA) is the ratio of pHRR to time to pHRR (TpHRR), the higher value of FIGRA means the rapid spread of fire and higher fire risk.^33^ The FIGRA of FR-silk sample decreased from 1.17 to 0.49 kW m^−2^ s^−1^ with 58.1% reduction. The results revealed that the modification was beneficial to endow silk fabric with excellent flame retardant properties.

**Table 3:** Cone calorimeter data of the control and FR-silk

Sample	TTI (s)	pHRR (kW m^−2^)	THR (MJ m^−2^)	TpHRR (s)	FIGRA (kW m^−2^ s^−1^)	Residue (wt%)
Silk	38	123.2	7.3	105	1.17	35.7
FR-silk	62	44.7	1.9	90	0.49	58

### 3.7. Flammability and washing durability

LOI test was used to evaluate the flame retardant performance of different samples before and after washing. The test results were listed in Table 4. The LOI values of pure silk, D-silk, PN-silk and FR-silk were 23.3%, 24.1%, 31.5% and 32.8% before washing, respectively. Compared to the control sample, the LOI values of D-silk, PN-silk and FR-silk increased by 18.8%, 35.5% and 40.7%, respectively. However, after 15 launderings cycles (LCs), the LOI values of D-silk, PN-silk and FR-silk decreased to 23.5%, 25.9% and 26.5%, respectively. The slight decrease in the LOI value of the D-silk was caused by the removal of the dye adsorbed on the surface of the fabric during the washing process. For PN-silk, because the ionic bond was generated when phosphoric acid and urea reacted with the amine groups on the silk fabric. Perhaps the low number of ionic bonds and weak bonding force caused the ionic bonds were lost during washing, which results in the effective flame retardant component, *i.e.*, the phosphoric acid group, was lost. Therefore, the flame retardant performance decreased sharply. For FR-silk, the effective flame retardant ingredient that adhered to the surface shed off from the silk mainly due to the strong mechanical friction and detergent dissolution effect. As a result, the LOI values of FR-silk decreased as the LCs increasing. But the LOI value of FR-silk was still higher than that of D-silk and PN-silk after 15 LCs. In general, ASY contributed to the flame retardant properties of silk fabrics, and FR-silk exhibited good washing resistance and was capable of meeting the demands of daily use.

**Table 4:** LOI values of different silk fabrics before and after different LCs

Sample	LOI values (%)			
	0 LCs	5 LCs	10 LCs	15 LCs
Silk	23.3	—	—	—
D-silk	24.1	23.8	23.6	23.5
PN-silk	31.5	29.7	27.0	25.9
FR-silk	32.8	30.8	27.7	26.5

**Fig. 8:**
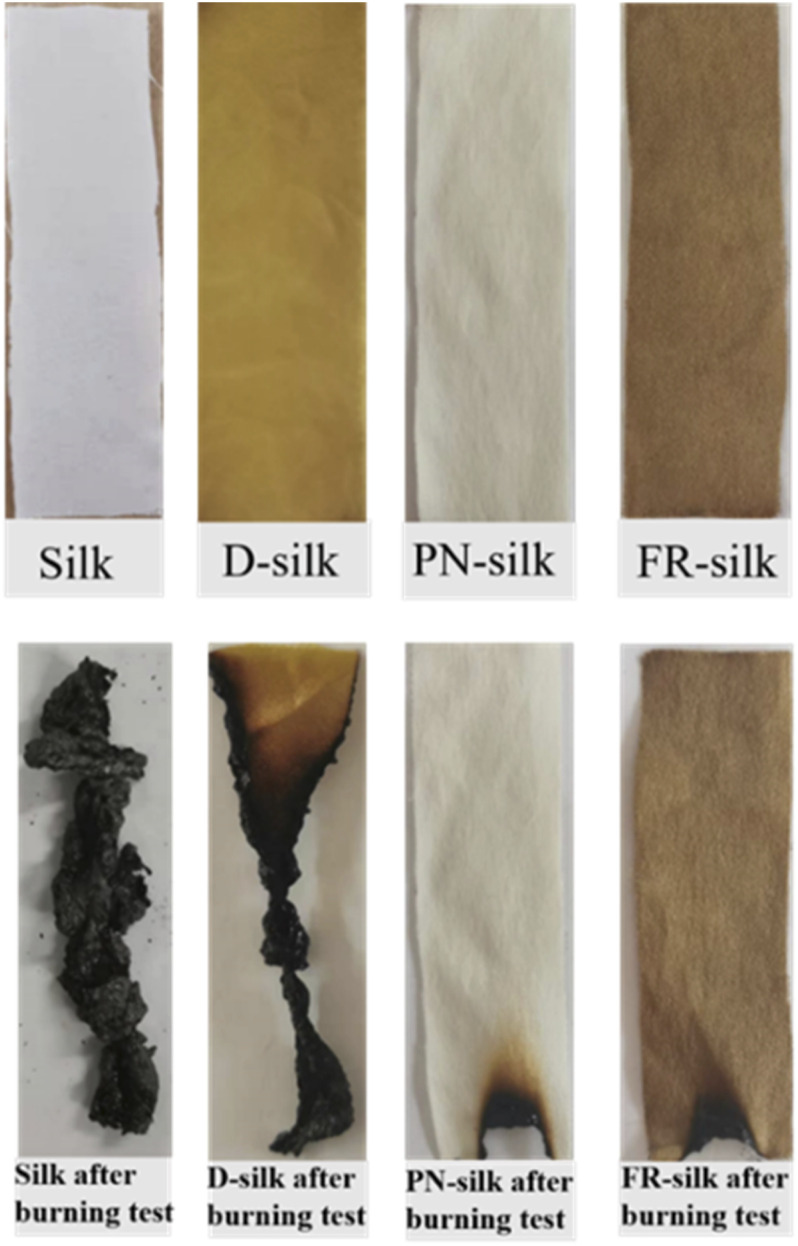
Photos of silk, D-silk, PN-silk and FR-silk after burning test.

For more display intuitive the flame retardant effect of the samples, the actual photos of samples before and burning test were shown in Fig. 8. Compared to the pure sample, PN-silk with only trivial color change. D-silk showed a bright, soft yellow color, while the yellow color of FR-silk was a bit darker than that of D-silk. After burning test, the blank sample was burned completely leaving light and brittle char residue. D-silk was partially burned in the burning test, and the char residue was as friable as the pure sample. Contrarily, PN-silk did not burn even if it was exposed to the flame for a long time, showing good flame retardancy. FR-silk also had excellent flame retardancy. The results indicated that the flame retardancy of D-silk was improved, but the flame retardancy of D-silk was far less than that of PN-silk and FR-silk fabric. It should be noted that although PN-silk had good flame retardancy, it had an absence of dyeing properties. In other words, the prepared ASY in the work can not only gave the fabric good flame retardant performance, but also impart the fabric dyeing functions.

### 3.8. Condensed phase analysis

**Fig. 9:**
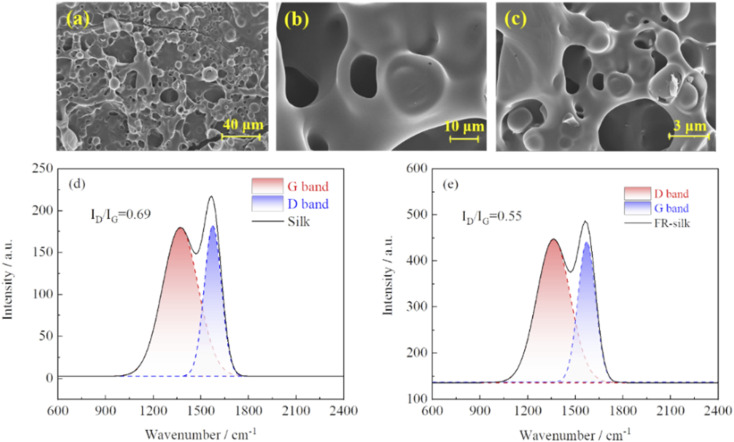
SEM images of the char residue of FR-silk (a–c); Raman spectra of the char residue of silk (d) and FR-silk (e).

As shown in Fig. 9a–c, FR-silk formed a highly expanded char layer with porous structure during combustion. The char layer could effectively prevent heat from transferring, protect the silk fiber matrix, and significantly improve the heat resistance and flame retardancy of silk fabric.^34^ The graphitization degree of the char residue of FR-silk was measured by Raman spectroscopy. As indicated in Fig. 9d and e, two peaks appeared at about 1347 and 1569 cm^−1^, ascribing to D-band of amorphous structure and G-band of graphite structure.^35^ In general, the ratio of the integral intensity of D and G band (*I*_D_/*I*_G_) was used to evaluate the graphitization degree. The lower ratio of *I*_D_/*I*_G_ indicated higher degree of graphitization, better shielding effect, better thermal stability, and higher stability of thermal oxidation.^36,37^ The calculated *I*_D_/*I*_G_ values of silk and FR-silk were 0.69 and 0.55, respectively. This demonstrated that FR-silk fabric exhibited a more complete char layer and fewer defects than pure fabric. In other words, the generated higher graphitized char could effectively insulate heat and oxygen, interrupt heat exchange and fuel supply, so as to achieve good flame retardant effect.

### 3.9. Gaseous phase analysis

**Fig. 10:**
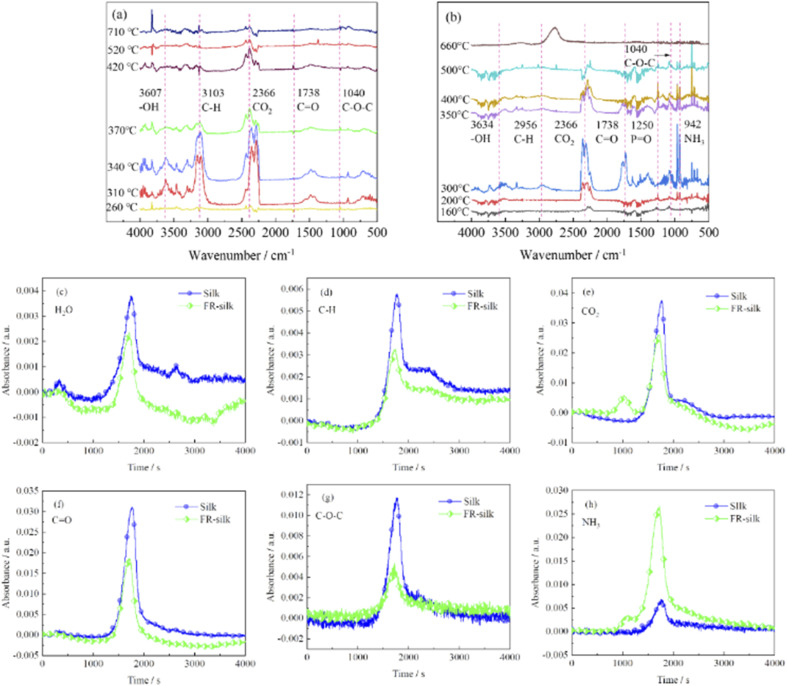
TG-IR spectra of silk (a) and FR-silk (b). The absorbance intensities of H_2_O (c), C–H (d), CO_2_ (e), C=O (f), C–O–C (g), and NH_3_ (h).

To further investigate the flame retardant mechanism of ASY on silk fabric, TG-IR was employed to detect volatile products derived from thermal degradation of the samples, and the corresponding results were shown in Fig. 10. The strong absorption peaks at 3500–4000 and 2366 cm^−1^ were attributed to vibrations of water vapor and carbon dioxide.^38,39^ For the treated samples, the initial release of water and carbon dioxide occurred at 160 °C, which was much earlier than that of the original sample (260 °C). This was mainly due to the accelerated dehydration and decomposition of silk fabric catalyzed by the phosphorus-rich components generating from the ASY pyrolysis. The absorption peak intensity of the pure sample of carbon dioxide was between 260 and 320 °C, and reached the maximum at 320 °C, then it was gradually decreased. However, the absorption peak of carbon dioxide of FR-silk appeared at 160 °C and its peak intensity was strongest at 300 °C. In addition, the intensity of the carbon dioxide absorption peak of the treated sample was weaker than that of the original sample, which was probably attributed to the dense and continuous char layer that inhibited the release of carbon dioxide. The characteristic peaks located at 1738, 1040 and 2956 cm^−1^ coincided with the C=O, C–O–C and C–H functional groups.^40,41^ These peaks of FR-silk appeared earlier and were more intense than those of the control sample. The new band at 1250 cm^−1^ appeared at 160 °C was mainly associated with the P=O group originating from ASY,^42^ which confirmed that phosphorus-containing radicals were generated during thermal degradation. These radicals could capture the HO˙ and H˙ radicals generated in the combustion zone and thus interrupt the chain reaction of combustion. The NH_3_ vibration peak was detected at 930 cm^−1^ and 942 cm^−1^ in the original and treated samples, respectively. It was worth mentioning that the absorption peak intensity of NH_3_ in the treated sample was much higher than that of the pure sample. This was conducive to diluting the combustibles concentration in the combustion zone and thus significantly delaying the ignition points of silk fabric.^41^ In summary, FR-silk released more non-toxic, non-flammable gases and phosphorus radicals derived from ASY, which played the flame retardant role in gas phase. In addition, the generated integrated and dense char residue catalyzed by phosphorus-containing acids played the role in condensed phase. In conclusion, the intensity of absorption peaks of C–H, C–O–C and C=O for FR-silk was decreased compared to the control silk, which illustrated that FR-silk released fewer combustible gases. In addition, for FR-silk, the release intensity of NH_3_ was remarkably higher compared to that of blank silk. The results demonstrated that FR-silk produced more non-combustible gases to play the role of gas phase flame retardant mechanism.

### 3.10. Flame-retardant mechanism analysis

**Fig. 11:**
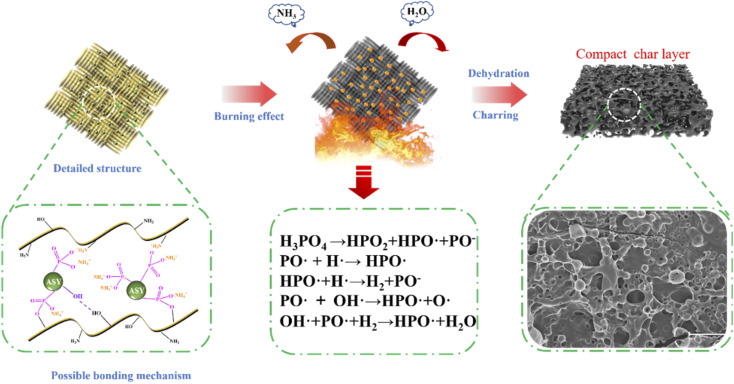
Possible flame-retardant mechanism for FR-silk.

Based on the above results, the possible flame retardant mechanism of FR-silk was proposed as depicted in Fig. 11. During modification, ASY could form covalent bond with hydroxyl group and ionic bond with amine group of the silk fabric. In addition, a large number of hydroxyl groups in ASY were conducive to the formation of hydrogen bonds with hydroxyl and amine groups in silk fabrics. These interactions ensured that the ASY could firmly bonded to the silk fabric. During combustion process, the ASY promoted the char-forming ability, forming a dense char layer on the fabric surface, and further acting as a physical barrier to protect the matrix material. In other words, the generated dense and integrated char residue played a heat insulator role in condensed phase. In gas phase, the active radicals (PO˙ and HPO˙) released by FR-silk during combustion captured the radicals of H˙ and HO˙ that promoted the chain growth reaction of the combustion.^43^ At the same time, FR-silk released some non-combustible gases such as CO_2_ and NH_3_, which diluted combustible gases and played the role in gas phase. Therefore, compared to the control silk, FR-silk was capable of reducing the release of combustible gases during combustion, while releasing a large amount of non-combustible gas. Overall, ASY combined with silk in a multi-binding manner and played a dual role in gas and condensed phase.

### 3.11. Color measurements and UV-resistant properties

To characterize the apparent color of silk fabric before and after modification, the modified silk with four layers were tested on DataColor 800. The results were presented in Table 5. In the CIELAB color space, the position of a color is usually represented by a 3D Cartesian coordinate system distinction. The degree of lightness and darkness of a color is indicated by the luminance value (L*). a* and b* represent the red and yellow areas of the CIELAB color space, respectively.^44,45^ The *K*/*S* values of FR-silk and D-silk were 5.9 and 5.7 respectively, and both gradually decreased with increasing LCs. The *K*/*S* value of FR-silk remaining at around 3.0 after 20 LCs. For D-silk, the color change during laundering was caused by the ionisation of the hydroxyl group on SY.^30^ On the one hand, the decrease in the *K*/*S* value of FR-silk may be due to the removal of ASY adsorbed on the surface of the silk fabric during the washing process. On the other hand, it is possible that the hydrogen bond between ASY and FR-silk was partially broken. The a* and b* values of D-silk remained positive after several low-speed runs (81.9, 4.3, 34.1). This meant that the color of D-silk was predominantly in the reddish-yellow area of the color space and that the D-silk fabric presented a bright, vivid and beautiful tone. After washing, the b* value of FR-silk decreased from 28.4 to 24.7. The b* value of FR-silk was always positive, although it decreased after washing. In addition, the L* value of FR-silk increased from 54.7 in the control sample to 65.6. The decrease in b* values and the increase in L* values for D-silk and FR-silk may be due to the fact that SY and ASY might alkaline in an alkaline wash environment. Compared to the blank sample, it was obvious that ASY was bonded to silk fabric, thus achieving flame retardancy and color fastness at the same time.

**Table 5:** Color measurement of D-silk and FR-silk before and after different LCs

Sample	*K*/*S*	L*	a*	b*
D-silk 0 LCs	5.7	73.2	6.4	39.7
D-silk 5 LCS	5.4	75.8	5.6	38.6
D-silk 10 LCs	4.8	81.4	4.8	38.2
D-silk 15 LCs	4.3	81.9	4.3	34.1
FR-silk 0 LCs	5.9	56.7	6.8	28.4
FR-silk 5 LCs	5.1	59.1	5.8	28.4
FR-silk 10 LCs	3.4	64.7	5.4	28.0
FR-silk 15 LCs	3.0	64.6	3.6	24.7

Detailed data on the washing color fastness of D-silk and FR-silk were displayed in Table 6. The color fastness of the fabrics is divided into 1–5 grades, and higher grade indicates better washing color fastness. The color fastness of the fabric is graded from 1–5, with higher grades indicating better wash fastness. Both D-silk and FR-silk were graded 4–5, indicating good color fastness of FR-silk to washing.

**Table 6:** Color fastness of D-silk and FR-silk

Samples	Color change	Staining on wool fabric	Staining on cotton fabric
D-silk	4–5	4–5	3–4
FR-silk	4–5	4	3–4

UV rays with wavelengths ranging from 100 nm to 400 nm are divided into UVC (100–280 nm), UVB (280–315 nm) and UVA (315–400 nm).^46^ Among them, UVB radiation was more pathogenic and harmful to humans and should be focused on protection. As shown in Table 7, UVA and UVB transmittance (%) and UV protection factor (UPF) of the samples were characterized. The UPF value, UVA and UVB transmittance (%) of the control silk were 15.26, 10.16 and 4.82, respectively, which were classified as insufficient protection. However, after the silk fabric treated by SY, *i.e.*, D-silk had enhanced UV protection with the UPF, UVA and UVB transmittance (%) of 6.57, 3.43 and 46.83, respectively, which was classified as good UV protection level. Compared to the pure sample, the UPF value of FR-silk was 73.79, which was dramatically higher than that of the blank sample and D-silk, in addition, the UVA and UVB transmittance (%) of FR-silk were obviously decreased to 1.58 and 1.27, respectively, belonging to excellent UV protection level.^47^ This was in accordance with the definition of “UV protection products” in GB/T18830-2002. The reason was that during higher temperature modification, silk fibers swelled and resulted in darker shades of the treated silk fabric, which accounted for the reduced UV transmittance.^48^ In addition, safflower yellow was a natural dye with inherent UV absorption effect.^49^ Therefore, the UV protection performance of FR-silk was greatly improved compared to the blank and D-silk sample, and it could be categorized as excellent UV resistant fabric according to GB/T 18830-2002.

**Table 7:** UV resistant properties of silk, D-silk and FR-silk

Samples	Transmittance (%)		UPF	Protection category
	UVA	UVB		
Silk	10.16	4.82	15.26	Insufficient protection
D-silk	6.57	3.43	46.83	Good protection
FR-silk	1.58	1.27	73.79	Excellent protection

### 3.12. Tensile strength of FR-silk

**Fig. 12:**
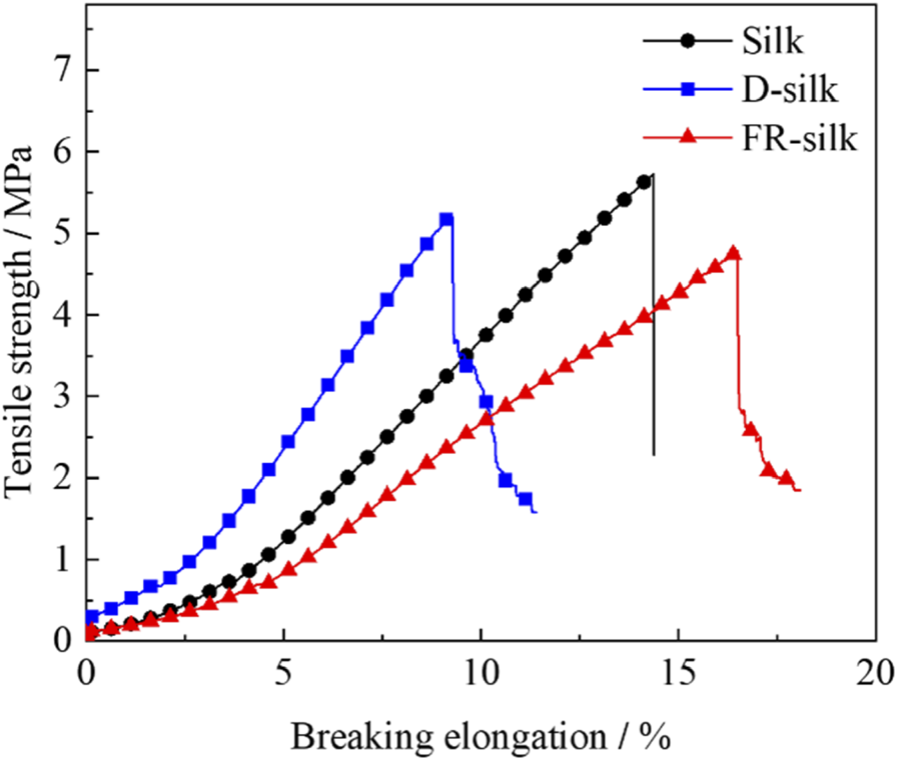
Mechanical performances of silk, D-silk and FR-silk.

It was necessary to evaluate the mechanical properties of silk fabrics after treatment so as to verify whether it has practical application. The curves were exhibited in Fig. 12. The tensile strength of D-silk and FR-silk were reduced slightly compared to that of the blank fabric. Compared to the control sample, the tensile strength of D-silk and FR-silk was decreased by 8.9% and 16.8%, respectively. For D-silk, the dyeing action between SY molecules and silk may destroy the regularity of molecules, weaken the intermolecular force of silk itself, and lead to the decrease of tensile strength.^50^ However, as for FR-silk, the reduced mechanical properties may be due to the hydrolysis of silk fibers under high temperature modification. In addition, flame retardant treatment solutions was weakly acidic, which might destroy the surface structure of silk fibers during the flame retardant dyeing modification. Furthermore, the dyeing process also affected the intermolecular force of silk, which was similar to that of D-silk. Overall, the flame retardant dyeing finishing had a negative impact on the mechanical properties of silk fabrics and resulted in the decrease of tensile strength. The reduction in tensile strength of the silk fabric was within acceptable limits and had little impact on the daily use of the silk fabrics.

## 4. Conclusions

In this work, silk fabrics with multifunctional of UV resistant, flame retardancy and dyeing were successfully prepared by a simple and effective one-pot method. FTIR, SEM and XPS indicated that the silk fabric was successfully modified. The TG results showed that the thermal stability of the treated silk was significantly improved. XPS illustrated that the percentage of P and N elements on the surface of the modified silk increased. The Raman and TG-IR results demonstrated that ASY played an important flame retardant role in gas and condensed phases. In addition, the modified fabric not only had good flame retardancy, excellent dyeing depth, washing color fastness and a certain brightness, but also had excellent UV resistant performance. This work provided a new method that was more environmentally friendly to impart silk fabric multifunction, such as flame retardancy, good color fastness and UV protection, which was facile and suitable for large-scale production.

## Author contributions

DanYu: formal analysis, investigation, resources, writing – original draft. Yan-song Liu: formal analysis, investigation, methodology, writing – original draft. Yuan-Lin Ren: conceptualization, resources, formal analysis, supervision. Xiao-Hui Liu: formal analysis, visualization, writing – review & editing. Hong-Qiang Qu: resources, methodology, visualization, supervision. Dan Yu and Yan-song Liu contributed equally to the work.

## Ethical statement

All authors state that they adhere to the Ethical Responsibilities of Authors. In addition, the work is compliance with ethical standards.

## Conflicts of interest

The authors declare that they have no known competing financial interests or personal relationships that could have appeared to influence the work reported in this paper.

## Supplementary Material
